# Dysregulated TP53 Among PTSD Patients Leads to Downregulation of miRNA let-7a and Promotes an Inflammatory Th17 Phenotype

**DOI:** 10.3389/fimmu.2021.815840

**Published:** 2022-01-04

**Authors:** Philip B. Busbee, Marpe Bam, Xiaoming Yang, Osama A. Abdulla, Juhua Zhou, Jay Paul (Jack) Ginsberg, Allison E. Aiello, Monica Uddin, Mitzi Nagarkatti, Prakash S. Nagarkatti

**Affiliations:** ^1^ Department of Pathology, Microbiology, and Immunology, School of Medicine, University of South Carolina, Columbia, SC, United States; ^2^ Departments of Psychophysiology, Clinical Psychology, and Research Office, Saybrook University, Pasadena, CA, United States; ^3^ Department of Epidemiology, University of North Carolina (UNC) Gillings School of Global Public Health, University of North Carolina, Mcgavran-Greenberg Hall, Chapel Hill, NC, United States; ^4^ Genomics Program, University of South Florida College of Public Health, Tampa, FL, United States

**Keywords:** post-traumatic stress disorder, tumor protein 53, microRNA, inflammation, T helper 17 cells

## Abstract

Post-traumatic stress disorder (PTSD) is a psychiatric disorder and patients diagnosed with PTSD often express other comorbid health issues, particularly autoimmune and inflammatory disorders. Our previous reports investigating peripheral blood mononuclear cells (PBMCs) from PTSD patients showed that these patients exhibit an increased inflammatory T helper (Th) cell phenotype and widespread downregulation of microRNAs (miRNAs), key molecules involved in post-transcriptional gene regulation. A combination of analyzing prior datasets on gene and miRNA expression of PBMCs from PTSD and Control samples, as well as experiments using primary PBMCs collected from human PTSD and Controls blood, was used to evaluate TP53 expression, DNA methylation, and miRNA modulation on Th17 development. In the current report, we note several downregulated miRNAs were linked to tumor protein 53 (TP53), also known as p53. Expression data from PBMCs revealed that compared to Controls, PTSD patients exhibited decreased TP53 which correlated with an increased inflammatory Th17 phenotype. Decreased expression of TP53 in the PTSD population was shown to be associated with an increase in DNA methylation in the *TP53* promotor region. Lastly, the most significantly downregulated TP53-associated miRNA, let-7a, was shown to negatively regulate Th17 T cells. Let-7a modulation in activated CD4+ T cells was shown to influence Th17 development and function, *via* alterations in IL-6 and IL-17 production, respectively. Collectively, these studies reveal that PTSD patients could be susceptible to inflammation by epigenetic dysregulation of TP53, which alters the miRNA profile to favor a proinflammatory Th17 phenotype.

## Introduction

Post-traumatic stress disorder (PTSD) is a psychiatric disorder caused by exposure to a severe traumatic event, such as combat, domestic violence, sexual assault, and natural disasters (1–3). In addition to classic psychiatric and psychologic symptoms of this disorder, such as emotional numbing, repetitive recollections of the traumatic event, and states of hyperarousal (4), other medical disorders have been linked to PTSD, including arthritis, diabetes, cardiovascular-associated conditions, and various inflammatory disorders (5–8). Increased risk of these comorbid medical disorders and medical complications in PTSD patients reduces the effective treatment of these patients, and studies have shown that PTSD patients have increased annual healthcare costs (4.2-9.3%) when compared to patients with other mental disorders (9). While the prevalence of PTSD was relatively low (8%) in the general population for a time (10), PTSD reported in combat veterans was found to be as high as 30% (11, 12), and reports are already showing increased prevalence of PTSD in numerous populations around the world due to the COVID-19 pandemic (13–15). Therefore, it is becoming increasingly critical to better understand why individuals diagnosed with PTSD have increased risk of other comorbid disorders. A better understanding of these mechanisms could provide both better treatment options and significantly improve the overall quality of life of the affected patient population.

Several reports link PTSD with increased inflammation and occurrence of inflammatory disorders (16–25). In an early report by Gola et al, researchers found that peripheral blood mononuclear cells (PBMCs) from PTSD patients exhibited a more pre-activated phenotype, having higher levels of secreting inflammatory cytokines, such as interleukin-6 (IL-6), tumor necrosis factor-alpha (TNF-α), and IL-1β when compared to control samples (26). We have since published extensively on the correlation between PTSD and inflammation, showing how epigenetic modifications (e.g. histone and DNA methylation) and modulation of microRNA (miRNA or miR), small noncoding RNA nucleotides involved in post-transcriptional regulation of genes, could potentially make PTSD patients more prone to inflammatory immune responses (27–31). Among these reports, published results revealed PBMCs from PTSD patients showed increased plasma IL-17 levels and CD4+ T helper-17 (Th17) phenotypes, which correlated with PTSD severity and global downregulation of miRNAs (22). Despite these observations, key regulators or promoters of this Th17 inflammatory phenotype in PTSD patients are yet to be fully understood.

In the current report, we noted several significantly dysregulated miRNA in PTSD PBMCs were associated with *TP53*, the human gene encoding for tumor protein 53 (TP53 or p53). Analysis of gene expression profile data in the public domain and our own evaluation of human PBMCs revealed TP53 is downregulated in PTSD subjects when compared to Controls. Whole genome methylated DNA immunoprecipitation sequencing (MeDIP-seq) data of PBMCs also showed that there was increased DNA methylation in the *TP53* promoter region of PTSD subjects compared to controls, which would suggest decreased transcription of this gene. Elsewhere in this report, we highlight that among the dysregulated miRNAs associated with TP53, let-7a was not only found to be the most significantly altered miRNA, but pathway analysis also revealed let-7a had direct and indirect links to Th17 cells. Lastly, transfection of activated CD4+ T cells purified from human PBMCs to modulate let-7a levels showed that this miRNA is directly involved in both Th17 development and function. The current report highlights an important role for the p53/miRNA(let-7a) axis in promoting a potential inflammatory Th17-skewed phenotype in the PTSD population.

## Materials and Methods

### Human Patient Samples

Procedures to collect human samples presented in this report were approved and reviewed by the Institutional Review Boards (IRBs) from the University of South Carolina, the University of Michigan, and the University of North Carolina-Chapel Hill. Blood sample collection from participants was performed after obtaining proper informed and written consent. In the current report, human samples were collected from two main sources: 1) veterans from the William Jennings Bryan Dorn Veterans Medical Center (VMC) (27), or 2) study participants from the Detroit Neighborhood Health Study (DNHS) (32). PTSD samples obtained from veterans at the William Jennings Bryan Dorn (VMC) were clinically assessed by medical VMC professionals using psychometric properties of the PTSD Checklist (PCL), Clinician Administered PTSD Scale (CAPS), and formal criteria from the Diagnostic and Statistical Manual of Mental Disorder (DSM-V), as previously described (29). For DNHS samples, PTSD diagnosis was performed using structured telephone interviews and the PTSD Checklist-Civilian Version (PCL-C), which was subsequently validated with in-person clinical interviews, as previously reported (32). The source and number of PTSD and Control samples were used for the following analyses present in the current report: A.) miRNA microarray evaluation of PBMCs: Control (n=4), PTSD (n=8); source: PBMCs from William Jennings Bryan Dorn VA participants; demographics data published in previous report (29). B.) TP53 and IL-17A expression from PBMCs from public database set: Control (n=16), PTSD (n=17); source: Gene expression profiles of PBMCs from combat veterans in public database Gene Expression Omnibus (GEO) database (ID GSE860); demographics data published in previous report (33). C.) TP53/Mdm2 PCR expression and correlation data from collected PBMCs: Control (n=6), PTSD (n=19); source: PBMCs from William Jennings Bryan Dorn VA participants; demographics data available in **Table 1**. D.) DNA methylation of the TP53 promoter region: Control (n=5); PTSD (n=5); source: PBMCs from DNHS project; demographic data available in **Table 1**. For Control samples, non-PTSD volunteers were found to be comparable to PTSD samples based on age, race, and sex, as detailed in **Table 1**. In addition, exclusion criteria for both the Control and PTSD samples included non-adult participants (< 18 years of age), participants with active infection(s) during PBMC collection, or other potentially immunocompromising conditions such as HIV, cancer, active pregnancy among female participants, or recorded history of chronic steroid use.

**Table 1:** Demographics and clinical history of Control and PTSD samples for PCR gene expression and DNA methylation analysis.

TP53 and Mdm2 Gene Expression (PCR)			
Parameters	Control (n = 6)	PTSD (n = 19)	P-value
Age	40.7 (7.3)	38.9 (9.4)	0.6173
Race			N/A
AA^a^	3 (0.5)	11 (0.6)	
CA^a^	3 (0.5)	6 (0.3)	
A^a^	0 (0.0)	1 (0.1)	
H^a^	0 (0.0)	1 (0.1)	
Sex			N/A
M^b^	5 (0.8)	17 (0.9)	
F^b^	1 (0.2)	2 (0.1)	
Depression score	13.3 (6.7)	31.4 (12.7)	0.0032**
Anxiety score	11.2 (11.2)	28.1 (12.7)	0.0078**
PTSD score	43.7 (3.6)	62.8 (12.1)	0.0010***
**For DNA Methylation of the TP53 Promoter**			
**Parameters**	**Control (n = 5)**	**PTSD (n = 5)**	**P-value**
Age	55.4 (18.0)	47.8 (17.4)	0.5162
Race			N/A
AA^a^	5 (1.0)	5 (1.0)	
CA^a^	0 (0.0)	0 (0.0)	
A^a^	0 (0.0)	0 (0.0)	
H^a^	0 (0.0)	0 (0.0)	
Sex			N/A
M^b^	4 (0.8)	2 (0.4)	
F^b^	1 (0.2)	3 (0.6)	

Values indicate the mean (standard deviation) for continuous variables (Age, Depression score, Anxiety score, PTSD score) and the number (proportion) was used for categorical variables (Race and Sex). Values were rounded to the nearest tenth. P-value was determined using unpaired two-tailed t test comparing Control and PTSD groups for continuous variable data (**P-value ≤ 0.01, ***P-value ≤ 0.001). Based on these results, Age, Race, and Sex were comparable between the two groups for the PCR data. Age and Race were comparable for the DNA methylation studies, though male proportions were skewed higher in Control samples, with higher proportions of females in the PTSD samples.

^a^AA, African American; CA, Caucasian; A, Asian; H, Hispanic.

^b^M, Male; F, Female.

N/A, not applicable.

### PBMC Isolation From Blood Samples

For PBMC isolation, researchers obtained deidentified blood samples (10-20 ml) collected in EDTA-coated tubes and began the isolation procedure within 1-2 hours of the sample collection. Blood samples were diluted in twice the volume of 1X PBS (Sigma-Aldrich, St. Louis, MO) before being layered on top of 20 ml of Ficoll-Paque (GE Healthcare, Uppsala, Sweden) and centrifuged at room temperature for 30 minutes at 1300 rpm. The upper plasma layer was carefully removed before collecting the PBMC interphase layer. PBMCs were diluted in twice the volume of 1X PBS before being centrifuged at 4°C for 5 minutes at 1300 rpm. Isolated PBMCs were washed twice in 1X PBS before further downstream processing of the cells (e.g. DNA/RNA isolation).

### miRNA Microarray and Analysis of PTSD and Control Samples

PTSD (n=8) and Control (n=4) samples were processed for miRNA microarray as previously reported (27). In the current report, significantly dysregulated miRNAs associated with direct or indirect links to TP53 were the major focus, thus preventing redundant or overlapping results from the previous report using this dataset. Briefly, total RNA was isolated from PBMC samples using the miRNeasy Mini kit (Qiagen, Valencia, CA), and RNA integrity was analyzed using an Agilent 2100 BioAnalyzer (Agilent Tech, Palo Alto, CA). Microarray was performed by the Deep Sequencing and Microarray Core Facility at John Hopkins Memorial Institute in Baltimore, MD. miRNA hybridization to the Affymetrix miRNA-v1 gene chip (ThermoFisher Scientific, Waltham, MA), microarray data normalization, microarray quality control, and calculation of linear fold change of miRNA between PTSD and Control samples were performed as previously reported (27). Ingenuity Pathway Analysis (Qiagen Valencia, CA), also known as IPA, was used to analyze significantly dysregulated miRNA in PTSD samples compared to Controls (defined as ± 1.5-fold change with p value of < 0.05) in relation to known and predicted gene targets. IPA was also used to identify and visualize dysregulated miRNA, target genes, and the potential interacting networks involved in reported or highly predicted functions (e.g. Diseases and Functions, Molecular and Cellular Functions). Raw miRNA microarray data is available at ArrayExpress (Accession# E-MTAB-4880).

### Evaluation of PTSD and Control Gene Expression Data From the Public Database

Initial analysis involving expression of genes of interest based on miRNA microarray data (e.g. TP53, Th17-related genes) was first evaluated in the public database, specifically from the NCBI (www.ncbi.nlm.nih.gov/) GEO database. The dataset used was ID GSE860 (33), which included normalized gene expression array data using the Affymetrix Human Genome U95A Array platform on PBMCs collected from PTSD (n=17) and Control (n=16) samples at two different time points (initial ER visit and 4 months after). Controls were defined as ER patients that experienced psychological trauma but did not develop PTSD over time. Normalized expression data was expressed as fold change relative to the average of gene expression of either Control or PTSD samples during the initial ER visit, prior to evaluation after 4 months. In the current report, results included evaluation of TP53 (31618_at) and IL-17A (1359_at) gene expression in the total of 33 PBMC samples included in the publicly available dataset, thereby reducing overlapping and repetitive results from the previous manuscript for which this data was associated with.

### Evaluation of Gene Expression in PTSD and Control Samples Using Quantitative Real-Time PCR

In addition to the aforementioned publicly available dataset, we evaluated gene expression in PBMCs from William Jennings Bryan Dorn VA participants between Control (n=6) and PTSD (n=19) groups for TP53 and the TP53 regulatory protein, Mdm2, using qRT-PCR. PBMC and RNA isolation procedures were performed from collected blood samples as described above. Complementary DNA (cDNA) was synthesized using miScript II RT kit (Qiagen, Valencia, CA), followed by gene transcript detection using iQ universal SYBR Green supermix (Bio-Rad Laboratories, Hercules, CA) on a Bio-Rad CFX384 PCR platform. A total of 10ng of cDNA was used per sample for the PCR reactions, and primers for TP53 (forward: CCTCAGCATCTTATCCGAGTGG; reverse: TGGATGGTGGTACAGTCAGAGC) and Mdm2 (forward: TGTTTGGCGTGCCAAGCTTCTC; reverse: CACAGATGTACCTGAGTCCGATG) were designed and created using Integrated DNA Technologies (IDT, Coralville, IA). Expression of genes were first normalized to 18S and then expressed as fold change using the delta-delta CT method (2^–ΔΔCt^ method). PCR reactions were performed using the following 2-step cycling protocol: initial denaturing and enzyme activation (95°C, 2:00 mins,1 cycle), denaturing (95°C, 15 secs, 40 cycles), annealing and extension (55°C, 30 secs, 40 cycles), melt curve (55-95°C with 0.5°C increments, 30 secs, 1 cycle). Control and PTSD participants used in these PCR assays and subsequent correlation studies were found to be comparable in terms of age, race, and sex.

### Methylated DNA Immunoprecipitation Sequencing of PTSD and Control PBMC Aamples

To investigate whole genome DNA methylation patterns, PBMCs were isolated from Control (n=5) and PTSD samples (n=5) provided from the DNHS project as described above. Genomic DNA was purified using a Zymo DNA purification kit (Zymo Research, Irvine, CA) and fragmented by Bioruptor sonicator (Diagenode, Denville, NJ). DNA fragments with sizes from 200 bp to 400 bp were purified and sequencing adaptors were added using Illumina Chip-seq library preparation kit. dsDNA was then denatured and immunoprecipitated with anti 5-methylcytosine antibody using a MeDIP kit from Diagenode. Precipitated DNA was purified and amplified. MeDIP-seq libraries were then sequenced by Nextseq550 with single-end reads. Raw sequencing reads were mapped to human genome build hg19 using Bowtie software by allowing two mismatches in the read (34). The mapped reads were then analyzed with MEDIPS software (35). Generated WIG files were visualized in the IGB genome browser (www.bioviz.org). Control and PTSD participants used in these in the DNA methylation studies were found to be comparable in terms of age and race. However, there were uneven distributions based on sex, with males being more proportionally represented in the Control group compared to females.

### MicroRNA let-7a Transfections in Activated and Purified CD4+ T Cell Cultures

PBMCs were isolated from blood of Control, non-PTSD volunteer samples as described above. PBMCs were purified for CD4 using an EasySep kit (StemCell Technologies, Cambridge, MA) and PE-conjugated monoclonal antibody (mAb) (Cat #555347, BD Biosciences, San Jose, CA). Purified CD4+ cells were assessed using FC500 flow cytometer (Beckman Coulter, Indianapolis, IN) and plated at 130,000 cell density per well in 24-well plates in complete RPMI media (Sigma-Aldrich, St. Louis, MO). Cells were left unactivated or activated with Dynabeads Human T-Activator CD3/CD28 (1:1 bead-to-cell ratio) (Cat. #111.61D, ThermoFisher Scientific, Waltham, MA) in 5% CO2 at 37 °C for 24 hours. In addition to activation, some cultures were transfected with either mock, Anti-hsa-let-7a-5p (inhibitor), or syn-has-let-7a-5 (mimic) from Qiagen (Valencia, CA). Transfections were performed using HiPerFect Transfection Reagent (Qiagen, Valencia, CA), following manufacturer’s instructions, with a final concentration of inhibitors and mimics at 20 µM. Mock cultures only contained transfection reagent without addition of either the mimics or inhibitors. After 24 hours, RNA was isolated from cultured cells to test let-7a transfection by PCR as described above. PCR was performed using miScript SYBR Green PCR kit (Qiagen, Valencia, CA) and human let-7a-specific primers using Qiagen-based miScript Primer Assay as per instructions from the manufacturer. In addition, Th17 cell phenotyping by flow cytometry using fluorescently-labeled mABs for CD4 (Cat #555347, BD Biosciences, San Jose, CA) and IL-17A (Cat# 555347, Biolegend, San Diego, CA) was performed. IL-17A intracellular staining was achieved using a BD Cytofix/Cytoperm solution kit as per manufacturer’s instructions (BD Biosciences, San Jose, CA). Cell culture supernatants were also assessed with human IL-17A and IL-6 ELISA kits from Biolegend (San Diego, CA).

### Statistical Analysis

Statistical analysis was performed using Graphpad Prism version 9 software. For matching Control and PTSD samples to be comparable with one another, continuous variables (age, PTSD scores, anxiety score, depression score) were expressed as means with standard deviations, and p-values calculated using an unpaired, two-tailed standard t test. For categorical variables (sex and race), these were expressed as numbers with overall proportions represented within each respective group (e.g. PTSD or Control) for qualitative comparisons. For other comparison data (e.g. miRNA fold change, PCR gene expression) between two groups, an unpaired, two-tailed standard t test was performed. When comparing 3 or more groups (e.g. *in vitro* cell culture studies with flow cytometry, miRNA fold change PCR, and ELISAs), one way ANOVA and *post-hoc* multiple comparisons Tukey’s test was performed. *In vitro* assays were performed at least three times and are shown as representative replicate data. For correlation studies, Pearson correlation tests were used to determine significance. Significance was defined as having a p value ≤ 0.05. For DNA methylation studies using Me-DIPseq, qualitative observations with intensity of signal (peak height and width) in the focused gene promoter regions (e.g. TP53) were evaluated as previously reported (28).

## Results

### Dysregulated miRNA in PTSD PBMCs Is Associated With TP53 and Inflammatory Th17 Response

**Figure 1:**
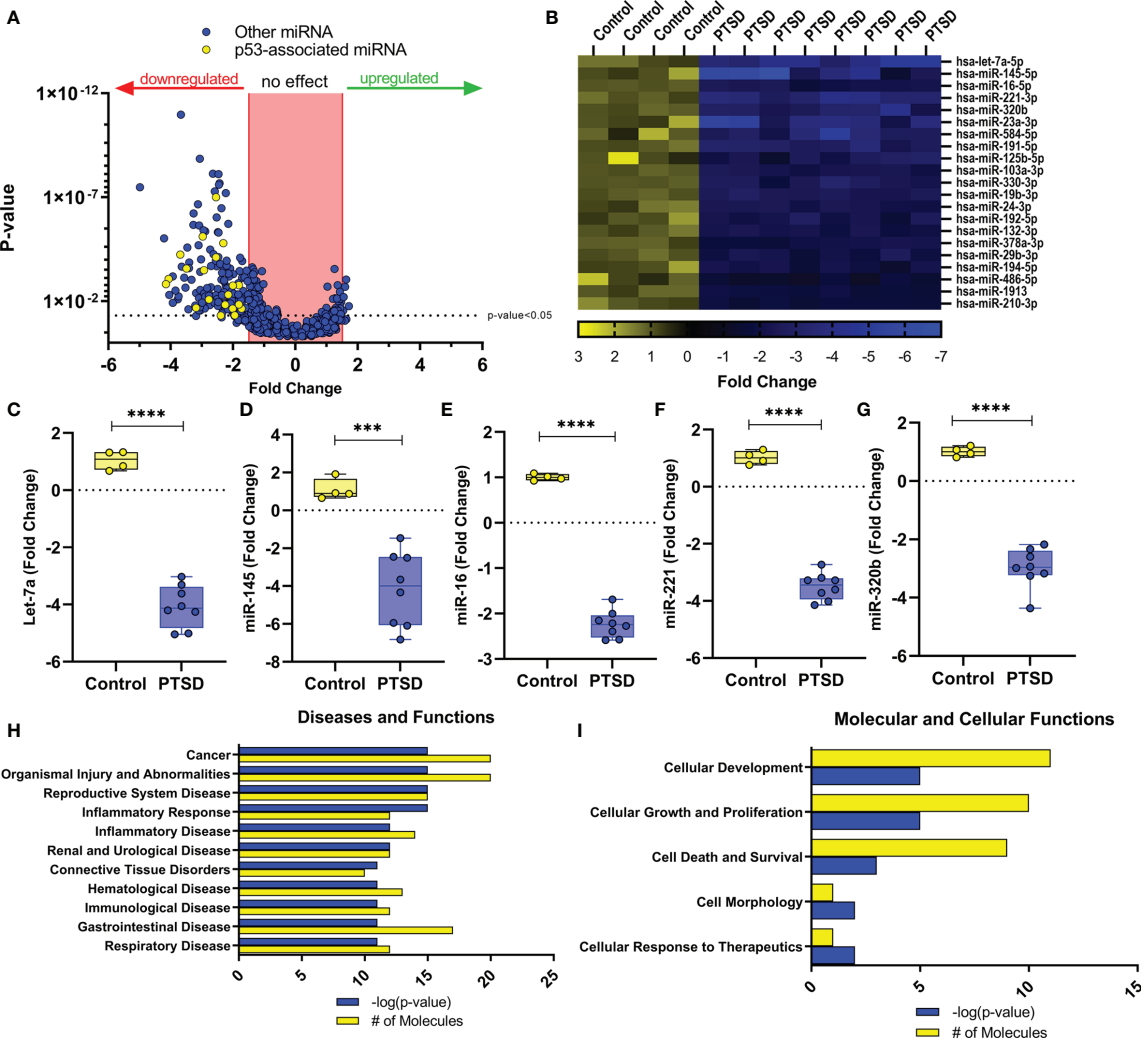
Dysregulated miRNA in PBMCs from PTSD patients are associated with TP53. miRNA array data from PBMCs from Control (n=4) and PTSD subjects (n=8) was analyzed using IPA analysis software. **(A)** Volcano plot depicting miRNA fold change values (x-axis) of PTSD vs. Control plotted against p-values (y-axis). Yellow dots represent TP53-associated miRNAs. **(B)** Heat Map depicting 21 TP53-associated miRNA that were significantly downregulated in PTSD samples compared to control. **(C–G)** Box and whisker plots depicting top 5 downregulated TP53-associated miRNA between Control and PTSD samples to include **(C)** let-7a, **(D)** miR-145, **(E)** miR-16, **(F)** miR-122, **(G)** miR-321b. Box and whisker bars depict maximum and minimum values, with middle bar depicted at the median. **(H, I)** Top ranked networks (as determined by IPA-derived –log p-values and # of molecules) for 21 total dysregulated miRNA associated with TP53 to include **(H)** Disease and Functions, as well as I) Molecular and Cellular Functions. Significance in linear fold change was determined as any value ≥1.5 or ≤ -1.5 fold, and p-value was determined significant if this value ≤ 0.05 using an unpaired standard t test. P-values: ≤ 0.005 (***), ≤ 0.001 (****).

**Figure 2:**
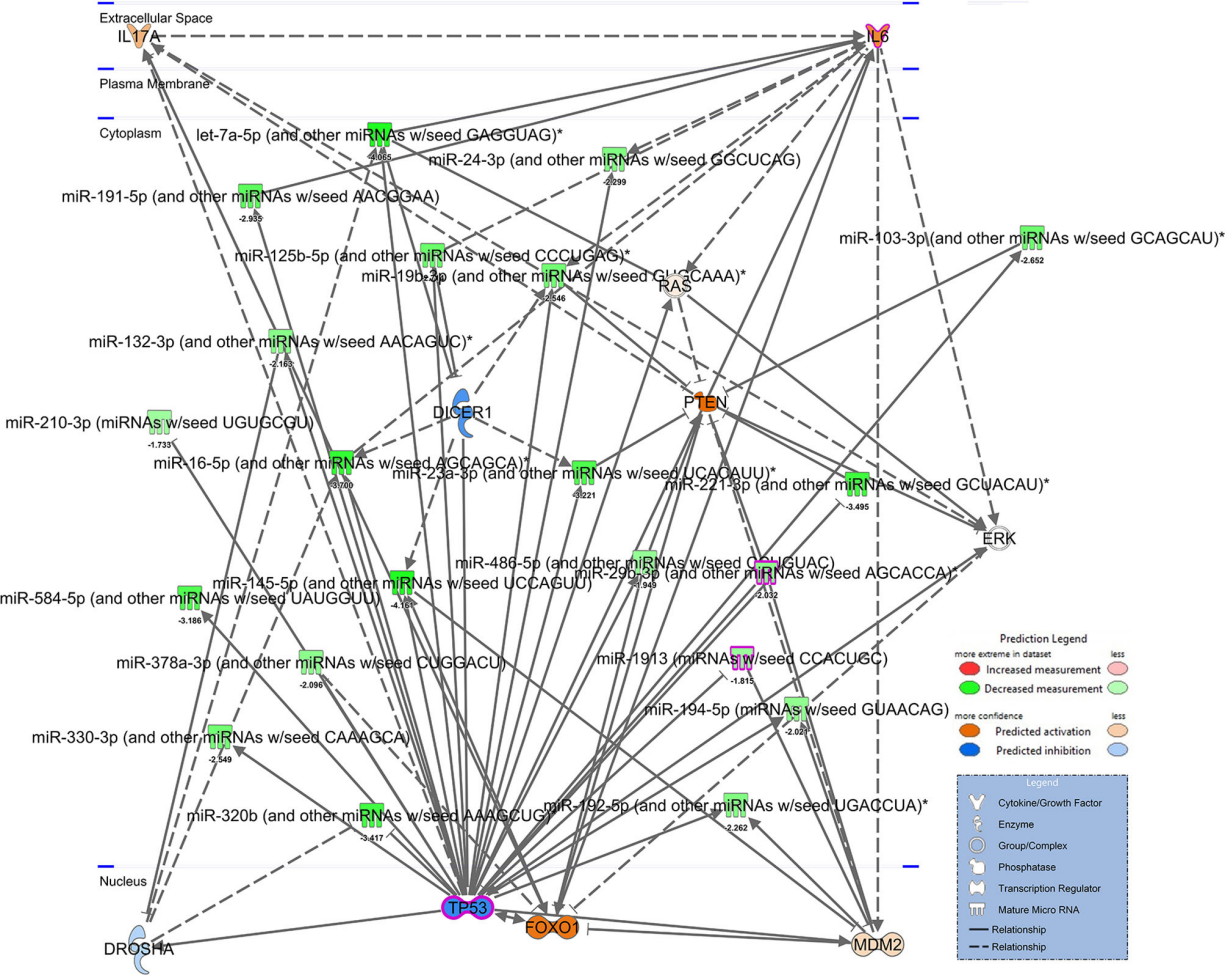
Network of TP53 interactions with dysregulated miRNA from PTSD patients. IPA-derived network depicting dysregulated miRNA associated with TP53 from mean fold changes of Control (n=4) vs. PTSD (n=8) PMBCs. miRNA are depicted in green to denote downregulation (darker green denotes more downregulation) or red to denote decreased expression. Genes depicted in the network are predicted to be activated (orange) or inhibited (blue) based on interactions present, as noted in the Prediction Legend. A separate legend (lower right corner) is provided to identify components depicted in the network. Solid lines indicate direct interactions, and doted lines indicate either indirect or highly-predicted interactions.

In our previous study, we noted that compared to healthy controls, PTSD patients had significant downregulation of a number of miRNA (27). Using IPA analysis software, we noted that of the top 6 networks affected by significantly dysregulated miRNA in PTSD samples, 4 of these included TP53 (**Table 2**). Further analysis revealed that of the 184 miRNAs significantly downregulated in PTSD samples, 21 of these altered miRNAS were found to be associated with TP53 (**Figures 1A** **, B**). The most significantly downregulated TP53-associated miRNA with the lowest p value was let-7a (**Figure 1C**), which was followed by miR-145, miR-16, miR-221, and miR-320b (**Figures 1D** **–G**). To better understand what processes and functions were affected by these particular miRNAs, only the 21 TP53-associated miRNAs were input back into IPA to determine how their alteration might affect disease and function. As shown in **Figure 1H**, Inflammatory Response and Inflammatory Disease were ranked within the top 5 Disease and Functions affected by these specific miRNA. In addition to Disease and Functions, the top Molecular and Cellular processes linked to these altered TP53-associated miRNAs included Cellular Development, as well as Cellular Growth and Proliferation (**Figure 1I**). Lastly, among the 21 TP53-associated miRNA, let-7a and TP53 were found to have direct and indirect interactions linked to Th17 development and function by way of their association with IL-6 and IL-17A, respectively (**Figure 2**). Prediction analysis based on the miRNA profiles also suggested that TP53 would be inhibited in PTSD samples, while IL-6 and IL-17A were predicted to be activated. These data suggested that through a TP53/let-7a axis, PTSD patients could be more prone to developing an abnormal Th17 phenotype.

**Table 2:** IPA-generated networks highlighting dysregulated miRNA and target genes between Control and PTSD samples.

Top Diseases and Functions Networks	Score	Focus Molecules	Molecules in Network
Cancer, Organismal Injury and Abnormalities, Reproductive System Disease	30	16	AGO2,BIRC5,DICER1,ETS1,JUN,miR-125b-5p (and other miRNAs w/seed CCCUGAG),miR-126a-3p (and other miRNAs w/seed CGUACCG),miR-128-3p (and other miRNAs w/seed CACAGUG),miR-130a-3p (and other miRNAs w/seed AGUGCAA),miR-139-5p (miRNAs w/seed CUACAGU),miR-150-5p (and other miRNAs w/seed CUCCCAA),miR-18a-5p (and other miRNAs w/seed AAGGUGC),miR-193a-5p (miRNAs w/seed GGGUCUU),miR-199a-5p (and other miRNAs w/seed CCAGUGU),miR-19b-3p (and other miRNAs w/seed GUGCAAA),miR-223-3p (miRNAs w/seed GUCAGUU),miR-23a-3p (and other miRNAs w/seed UCACAUU),miR-361-5p (miRNAs w/seed UAUCAGA),miR-487b-3p (miRNAs w/seed AUCGUAC),miR-494-3p (miRNAs w/seed GAAACAU),miR-96-5p (and other miRNAs w/seed UUGGCAC),SSB,TNRC6B, ***TP53***
Cancer, Hematological Disease, Immunological Disease	29	15	AGO2,AQP4,miR-103-3p (and other miRNAs w/seed GCAGCAU),miR-151-3p (and other miRNAs w/seed UAGACUG),miR-151-5p (and other miRNAs w/seed CGAGGAG),miR-188-3p (miRNAs w/seed UCCCACA),miR-1913 (and other miRNAs w/seed CUGCCCC),miR-320b (and other miRNAs w/seed AAAGCUG),miR-342-3p (miRNAs w/seed CUCACAC),miR-342-5p (and other miRNAs w/seed GGGGUGC),miR-378a-3p (and other miRNAs w/seed CUGGACU),miR-421-3p (and other miRNAs w/seed UCAACAG),miR-423-5p (and other miRNAs w/seed GAGGGGC),miR-455-3p (miRNAs w/seed CAGUCCA),miR-501-3p (and other miRNAs w/seed AUGCACC),miR-532-3p (miRNAs w/seed CUCCCAC),miR-668-3p (and other miRNAs w/seed GUCACUC),RPS15,SSB, ***TP53***
Cancer, Inflammatory Disease, Inflammatory Response	29	17	BCL6,CDC25C,CDKN2A,EIF4E,FAM3C,KLF4,LAMP2,let-7a-5p (and other miRNAs w/seed GAGGUAG),mir-9,mir-130,mir-146,miR-1246 (miRNAs w/seed AUGGAUU),miR-125b-5p (and other miRNAs w/seed CCCUGAG),miR-1275 (and other miRNAs w/seed UGGGGGA),miR-140-3p (and other miRNAs w/seed ACCACAG),miR-145-5p (and other miRNAs w/seed UCCAGUU),miR-194-5p (miRNAs w/seed GUAACAG),miR-200b-3p (and other miRNAs w/seed AAUACUG),miR-210-3p (miRNAs w/seed UGUGCGU),miR-29b-3p (and other miRNAs w/seed AGCACCA),miR-30c-5p (and other miRNAs w/seed GUAAACA),miR-324-5p (miRNAs w/seed GCAUCCC),miR-330-3p (and other miRNAs w/seed CAAAGCA),miR-339-5p (and other miRNAs w/seed CCCUGUC),miR-486-5p (and other miRNAs w/seed CCUGUAC),miR-503-5p (miRNAs w/seed AGCAGCG),miR-532-5p (and other miRNAs w/seed AUGCCUU),MYC,PPM1D,RBPJ,SSB,TOP2A, ***TP53***,YBX1,ZFP36L1
Cancer, Organismal Injury and Abnormalities, Reproductive System Disease	22	14	AGO2,CCND3,CHEK1,Creb,CXCL12,CYCS,DICER1,KAT6A,KIF23,MET,mir-130,miR-132-3p (and other miRNAs w/seed AACAGUC),miR-16-5p (and other miRNAs w/seed AGCAGCA),miR-185-5p (and other miRNAs w/seed GGAGAGA),miR-191-5p (and other miRNAs w/seed AACGGAA),miR-192-5p (and other miRNAs w/seed UGACCUA),miR-194-5p (miRNAs w/seed GUAACAG),miR-210-3p (miRNAs w/seed UGUGCGU),miR-22-3p (miRNAs w/seed AGCUGCC),miR-221-3p (and other miRNAs w/seed GCUACAU),miR-24-3p (and other miRNAs w/seed GGCUCAG),miR-330-3p (and other miRNAs w/seed CAAAGCA),miR-331-3p (miRNAs w/seed CCCCUGG),miR-486-5p (and other miRNAs w/seed CCUGUAC),miR-584-5p (and other miRNAs w/seed UAUGGUU),PLK1,PRC1,PTK2,PTPN1,RHOA,SSB, ***TP53***,YBX1,ZEB1,ZYX
Dermatological Diseases and Conditions, Cancer, Endocrine System Disorders	16	11	CARD11,CXCL3,CXCL8,DMBT1,estrogen receptor,FCER2,IL1RAP,IL1RL2,IL23A,IL36A,IL36B,IL36G,KRAS,LTF,mir-146,miR-143-3p (and other miRNAs w/seed GAGAUGA),miR-146a-5p (and other miRNAs w/seed GAGAACU),miR-148a-3p (and other miRNAs w/seed CAGUGCA),miR-155-5p (miRNAs w/seed UAAUGCU),miR-181a-5p (and other miRNAs w/seed ACAUUCA),miR-21-5p (and other miRNAs w/seed AGCUUAU),miR-26a-5p (and other miRNAs w/seed UCAAGUA),miR-27a-3p (and other miRNAs w/seed UCACAGU),miR-31-5p (and other miRNAs w/seed GGCAAGA),miR-491-5p (and other miRNAs w/seed GUGGGGA),miR-708-5p (and other miRNAs w/seed AGGAGCU),NFkB (complex),PELI1,PTAFR,S100A12,TIMP3,TLR1,TLR3,TLR10,VHL
Cell Cycle, Cancer, Cellular Development	7	6	AGO2,BCL2L1,BCL2L11,BIRC5,BMP2,CCND1,CDKN1B,CHUK,DICER1,EIF4E,EIF4EBP1,GSK3B,H2AFX,IGF1,KDM5B,KRAS,let-7a-5p (and other miRNAs w/seed GAGGUAG),MAP2K1/2,Mek,miR-100-5p (and other miRNAs w/seed ACCCGUA),miR-17-5p (and other miRNAs w/seed AAAGUGC),miR-193a-3p (and other miRNAs w/seed ACUGGCC),miR-199a-3p (and other miRNAs w/seed CAGUAGU),miR-92a-3p (and other miRNAs w/seed AUUGCAC),MTOR,MUC1,p70 S6k,PTEN,RPS6,RPTOR,Smad2/3,SRC,STAT3,TGFBR2,VIM

The ranking of Disease and Function Networks is based on the score and number of molecules involved in the pathways associated with significantly altered miRNA between Control (n=4) and PTSD (n=8) samples. TP53 (in 4 of the top 6 Networks) has been put in bold and italic text.

### TP53 Downregulation in PTSD PBMCs Correlates With Increased Inflammatory IL-17A

**Figure 3:**
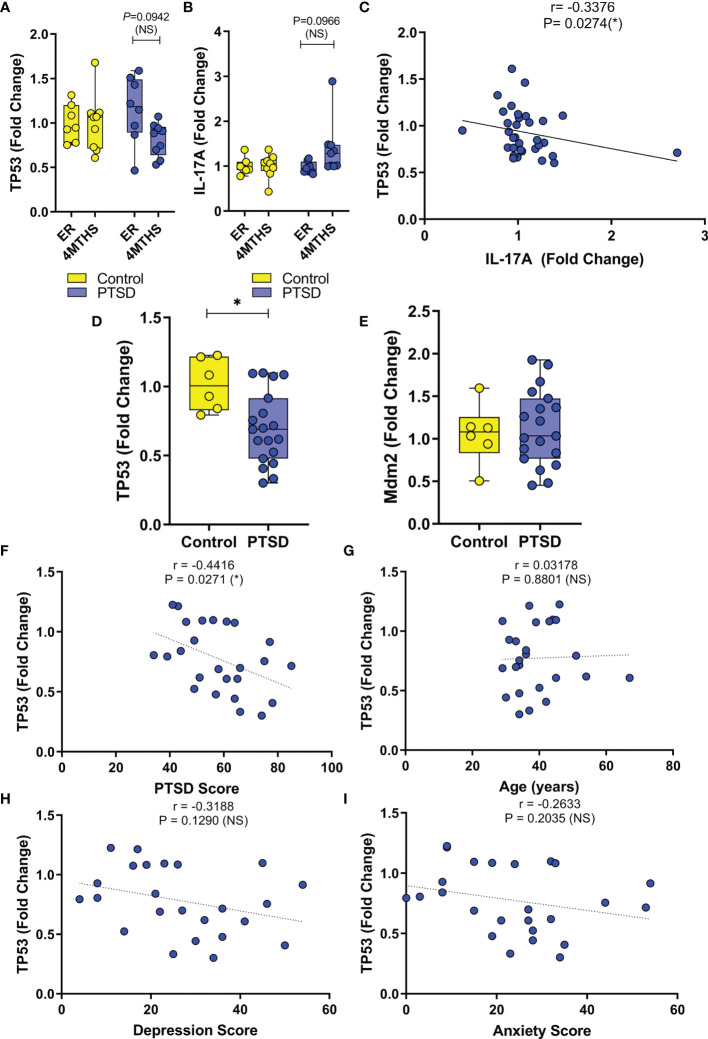
Expression of TP53 in PBMCs is downregulated in PTSD samples compared to Control. **(A–C)** Normalized expression data was analyzed from public database (ID. GSE860) between PTSD (n=17) and Controls (n=15), with two time points during initial emergency room visit for trauma (ER) and 4 months later (4MTHS). **(A)** Box and whisker plot depicting fold change expression of TP53. **(B)** Box and whisker plot depicting fold change expression of IL-17A. **(C)** Correlation plot between TP53 and IL-17A fold change expression. **(D–I)** PBMC gene expression data was collected from VMC PTSD (n=19) and Control volunteers (n=6). **(D)** Box and whisker plot depicting TP53 expression by PCR. **(E)** Box and whisker plot depicting Mdm2 expression by PCR. Correlation plots depicting **(F)** TP53 vs. PTSD score, **(G)** TP53 vs age of cohort, **(H)** TP53 vs. Depression score, and **(I)** TP53 vs. Anxiety score. Box and whisker bars depict maximum and minimum values, with middle bar depicted at the median. For box and whisker plots, significance was determined using an unpaired standard t test. For correlation plots, Pearson correlation test was used to determine significance. Significance was defined as having p-value ≤ 0.05 (*).

Since so many downregulated miRNA in PTSD samples were linked to TP53, initial evaluation of TP53 expression was undertaken in the public database. In database GSE860, normalized expression of gene data was available between PTSD and Control samples during two major time points: 1) a visit to the ER caused by trauma, and then 2) 4 months after their initial ER visits. Control subjects were deemed to not develop PTSD after the 4-month period. Control and PTSD subjects were found to have no significant differences in age, race, or sex, as detailed in the previous published report (33). As the data shows, when compared to Control samples, PBMCs collected from PTSD subjects exhibited a decrease in TP53 4 months after trauma was experienced, which trended towards significance (**Figure 3A**). In addition, it was interesting to note that in this same cohort of PTSD subjects, IL-17A expression had a noticeable trending increase after 4 months (**Figure 3B**). While both these observations trended towards significance, data showed there was in fact a significant negative correlation between TP53 and inflammatory cytokine IL-17A levels (**Figure 3C**). In addition to this publicly available data, we evaluated TP53 expression in PBMCs collected from local veterans at the William’s Jennings Bryan VMC. In this study, we collected PBMCs from both PTSD (n=19) and Control volunteers (n=6), with demographic data supplied in **Table 1**. Control and PTSD participants matched comparatively in terms of age, race, and sex, with no noted significant differences among these criteria between the two groups. There was however a larger overall proportion of male to female participants in this particular study, due to limited numbers of both Control and PTSD female volunteers. However, we found that veterans diagnosed with PTSD had significantly lower expression of TP53 compared to Controls (**Figure 3D**). Interestingly, there was no significant difference in the expression of TP53-inhibiting protein Mdm2 (**Figure 3E**), suggesting that TP53 expression was being specifically altered in the PTSD group, independent of other confounding factors or regulatory mechanisms related to TP53. Indeed, based on these studies, there was a clear significant negative correlation between TP53 expression and PTSD severity (**Figure 3F**), inasmuch as when the TP53 expression decreased, PTSD scores increased among the sampled population. Additionally, even though PTSD samples in this particular cohort had significantly higher anxiety and depression scores compared to Controls (**Table 1**), TP53 expression appeared to only be correlated with PTSD score, as there was no significant correlation between TP53 expression and the two commonly comorbid psychological conditions associated with PTSD (**Figures 3G** **–I**). Taken together, these data revealed that when compared to Controls, PBMCs from persons diagnosed with PTSD had lower expression of TP53.

### PBMCs From PTSD Subjects Exhibited Increased Methylation in One of the Known Promoter Regions of TP53

**Figure 4:**
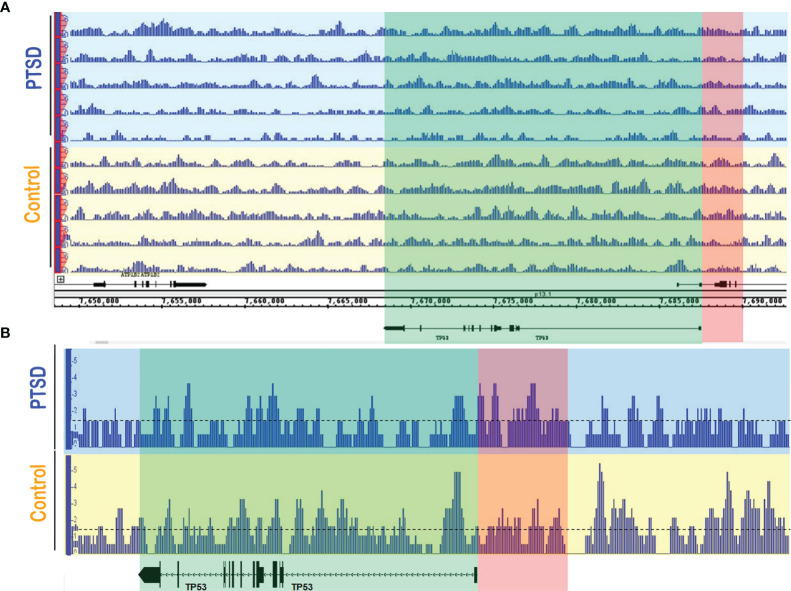
PTSD PBMCs had increased DNA methylation in TP53 promoter region. MeDIP-seq was performed on randomly selected PTSD (n=5) and Control (n=5) PBMC samples. Visualization of the methylated genome was acquired in the IGB genome browser (www.bioviz.org) for **(A)** individual samples, and **(B)** combined samples within respective groups. Blue sections indicate PTSD samples, and yellow sections represent Controls. Green section indicates the TP53 gene, while the red section represents one of the known TP53 promoter regions (~5kb) provided in the Ensembl genome browser (www.ensembl.org). For combined data, a line at 1.5 value was drawn to better visualize methylation intensity between the two groups. Differences observed are qualitative measures in which increased signal intensity (e.g. peak height and width) signify more DNA methylation. Signal intensity in the PTSD group was greater overall in the TP53 promoter compared to Control samples.

As we already noted that DNA methylation in genes involved in inflammation were altered in PTSD subjects (29), we evaluated the methylation pattern of the *TP53* promoter regions in Control (n=5) and PTSD (n=5) PBMC samples obtained from the DHNS project using MeDIP-seq. Patient demographics showed Control and PTSD samples used were comparable to each other in terms of age, race, and sex, though there was a slight disproportionate number of males in the Control samples (**Table 1**). This more male sex bias proportion was also present when evaluating Control and PTSD TP53 expression in the current report, as noted earlier. The samples collected in the DNHS project were also all African American (AA), which correlated with a larger proportion of this ethnicity in the aforementioned studies evaluating TP53 expression among PTSD samples, at least making the DNA methylation results more comparable to the aforementioned PCR results. In assessing one of the two identified promoter regions of the human *TP53* gene on the Ensembl genome browser (ensembl.org), it was found that in general, PTSD PBMC samples had increased methylation intensity in the larger ~5kb promoter region identified in **Figure 4A**. This increased DNA methylation intensity was even more noticeable when combining the 5 samples into their respective groups (**Figure 4B**). With increased DNA methylation in the promotor region, it would stand to reason that this would lead to an impediment in the active transcription of TP53, or the gene encoding the p53 protein. This epigenetic modification could explain why PBMCs from PTSD subjects have lower expression of TP53 when compared to healthy controls.

### Modulation of Let-7a in Activated CD4+ T Cells Alters Th17 Development and Function

**Figure 5:**
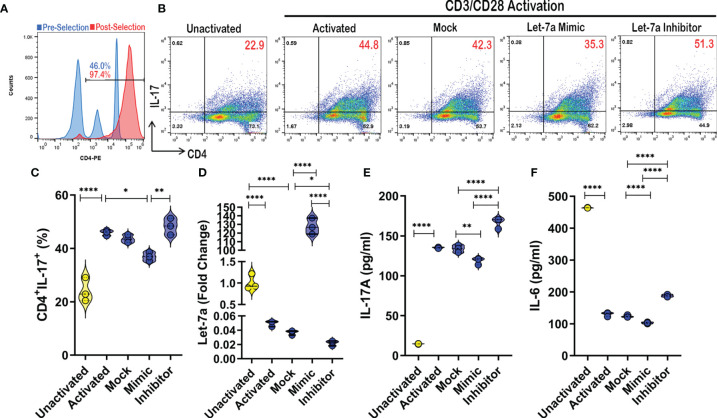
Let-7a modulation affects Th17 development and function. **(A)** CD4+ cells were purified using magnetic bead separation from PBMCs provided by healthy control donor, which was validated using flow cytometry to ensure purity (>95%). **(B)** Representative flow cytometry plots depicting Th17 cells (CD4+IL-17A+) in unactivated or CD3/CD28 activation cultures (24 hours) in the absence or presence of mock, let-7a mimic, or let-7a inhibitor. Red numbers indicating double positive TH17 percentage. **(C)** Violin plot depicting Th17 percentages as assessed by flow cytometry. **(D)** Violin plot depicting expression of let-7a as assessed by PCR. **(E)** Violin plot depicting IL-17 concentration as assessed by ELISA from cell culture supernatants. **(F)** Violin plot depicting IL-6 concentration as assessed by ELISA from cell culture supernatants. *In vitro* data are representative of at least 3 independent experiments, with 3 samples per culture condition. One way ANOVA was used to determine significance between groups. Significance was determined as p-value ≤ 0.05(*), ≤ 0.01(**), ≤ 0.001(****).

As it has already been experimentally shown that TP53 modulation affects let-7a expression (36, 37), experiments were carried out focusing on how let-7a alterations affected CD4+ T cell phenotypes during activation. To achieve this, CD4+ cells were purified (~97% consistently) from PBMCs from a Control samples (**Figure 5A**). Next, these CD4+ cells were either left unactivated, or activated with CD3/CD28 antibodies in the presence or absence of let-7a mimic or inhibitor. Because IPA miRNA analysis pathway linked let-7a to Th17 development and function (**Figure 2**), Th17 cells (CD4+IL-17A+) were assessed under these conditions using flow cytometry (**Figure 5B**). As expected, Th17 populations expand under activating conditions when compared to cells left unactivated, but what was interesting was that the Th17 populations were altered by let-7a modulation (**Figure 5C**). Specifically, when enhancing let-7a levels with the mimic, Th17 percentages significantly decreased, while the opposite was true as inhibiting let-7a led to expansion of the Th17 population in the cultures. It is noteworthy that the activation of these CD4+ cultures resulted in significant decreased expression of let-7a in general when compared to unactivated cells. Let-7a modulation was validated by PCR to show that the mimics significantly enhanced let-7a expression, whereas inhibitors significantly decreased the expression in these cultured conditions (**Figure 5D**). Once confirmed, Th17 function and development were evaluated by way of IL-17A and Th17-inducing IL-6, respectively. It was shown that under these culturing conditions, increases in let-7a led to a significant decrease in IL-17A production (**Figure 5E**). Inhibiting let-7a however promoted significant increased secretion of IL-17A. The same trend was observed in IL-6 production levels, where inhibition of let-7a significantly enhanced IL-6 secretion, but a decrease in this miRNA resulted in significantly increased IL-6 production (**Figure 5F**). Taken altogether, these data demonstrated that let-7a negatively regulates Th17 development and function. Combined with the previous results, the data suggested that decreased TP53 correlates with decreased biogenesis of let-7a in PTSD patients, promoting an increased Th17 phenotype, which would normally be regulated by miRNA let-7a. The overall conclusions suggested that patients diagnosed with PTSD may be more prone to an inflammatory Th17 phenotype due to dysregulation of the P53/let-7a axis, which under normal conditions would aid in regulating this T cell-mediated inflammatory response.

## Discussion

PTSD is a psychiatric disorder characterized by exposure to profound traumatic events that result in acute (less than 3 months), chronic (3 months or more), or delayed (occurring months or years after event) onset. Core symptoms are reminders that trigger memories of traumatic events, suppression of memories of the traumatic events, recurrence of disturbing flashbacks, avoidance or emotional numbing, and hyperarousal (4). In addition to these negative impacts on mental health, research has revealed a profound alteration of the immune system in patients with PTSD, which results in a higher risk for developing autoimmune and inflammatory disorders (16). During stressful events, the hypothalamic-pituitary-adrenal (HPA) axis is mobilized, which can activate the sympathetic nervous system to release adrenergic hormones (38). All lymphocytes express adrenergic receptors in varying degrees, including T cells (39, 40). In conjuncture with this notion that stress can have an impact on T cells, research has shown that people suffering from PTSD have increased T cell number and enhanced T cell function (6, 41–43). However, it is also important to note conflicting reports have also shown reduced T cell number and function in some cases of PTSD (44–46). Interestingly, in the cases reporting a reduction, these studies examined non-combat PTSD subjects, while cases showing an increase were from combat-associated PTSD. In this report and our previous report, we noted an increased inflammatory T cell response, but once again, this was observed more in veteran patients (27). Therefore, the type of trauma-inducing PTSD may play a significant role in T cell modulation outcome, and thus should be taken into consideration. More importantly, studies examining PTSD should also take into account certain cell types and not just general PBMCs, particularly in the case of CD4+ T cells, which have pro- and anti-inflammatory roles.

Generally, CD4+ T helper (Th) cells can be divided into pro- (Th1 and Th17) and anti- (Th2 and Treg) inflammatory subsets (47). In our previous report examining CD4+ T helper subsets in PTSD patients, we found that PTSD subjects had increased proportions of pro-inflammatory (Th1 and Th17) and reduced (Treg) or unchanged (Th2) anti-inflammatory CD4+ T cell subsets (27). In support of our findings, Gola and colleagues also showed PBMCs from PTSD patients spontaneously produced higher levels of IL-6, one of the key cytokines involved in Th17 differentiation (26). In our previous reports, we also provided possible mechanisms leading to the development of an increased inflammatory phenotype, which include miRNA modulation and epigenetic modifications to inflammatory-related genes and pathways, such as the Wnt signaling pathway (31). Building upon these results, we also noted an interesting association of PTSD with TP53 in the current study.

Our highlighted mechanism involving TP53 dysregulation in PTSD subjects is both supported and contradicted by other reports analyzing gene expression profiles of PBMCS from PTSD patients (48, 49). In the report from Dong et al., researchers noted TP53 was the most dysregulated gene in PTSD patients when compared to control samples, but interestingly the expression was elevated (48). On the other hand, in support of our current findings, Breen et al. noted that TP53 was downregulated in PTSD-positive samples (49). The discrepancy once again might be better explained with more careful analysis in the future, one that takes into account the type of trauma and cell type, admittedly a limitation even in the current report. In particular, it will be crucial in future studies to validate many of these findings (e.g. TP53 expression and DNA promoter methylation, let-7a levels) in purified CD4+ T cell populations from PTSD and control samples, as opposed to whole circulating PBMC populations, which consists of a variety of different immune cell types. However, it is interesting to take in consideration what we currently know about TP53 and how it regulates the Th17 inflammatory response. TP53 has not only been shown to negatively regulate the function and differentiation of Th17 effector cells (50), but has also been shown to play a role in the biogenesis of several miRNA (51), small non-coding RNA molecules known to regulate several cellular functions including the development of Th17 cells (52). In response to DNA damage or some abnormal oncogene activation, TP53 will act as a tumor suppressor by preventing tumor growth and development through several pathways including apoptosis, senescence, and cell cycle arrest (53). There are an abundant number of resources and research articles dedicated to the role TP53 plays in cancer and tumor development, but more studies are focusing on the role this protein plays in normal cellular processes, particularly in terms of T cell function. Watanabe and colleagues showed that TP53 plays an important role in antigen-specific CD4+ T cell responses, and proliferation of antigen-activated CD4+ T cells was dependent on the downregulation of TP53 (54). TP53 was also found to induce SLAM-associated protein (SAP), a pro-apoptotic factor in lymphocytes, in activated primary human T cells as a means to maintain T cell homeostasis (55). More importantly, in a murine model investigating the effects of autoimmunity in p53null CD45.1 mice, these mice were shown to spontaneously develop autoimmunity, characterized by increased presence of Th17 effectors, as well as elevated serum levels of IL-17 and Th17-producing IL-6 (50). Collectively, these data suggested that TP53 is not only a major player in T cell development and function, but it may also support our current findings suggesting that the TP53/let-7a axis may also negatively regulate Th17 CD4+ subsets, warranting further investigation into the role this protein might be play in the Th17-driven immune response in the PTSD population.

It is important to note that other cell types can produce IL-17A, such as γδ T cells, innate lymphoid type cells (ILCs), mast cells, CD8 T cells, natural killer (NK) cells, and neutrophils (56, 57). However, the source of increased IL-17A observed in PTSD patient PBMCs relative to controls in our previous report (27) and highlighted in the current report is more than likely produced by Th17 cells specifically. IL-17A was co-expressed with either CD4 or a combination of CD3 and CD4. This would exclude cytotoxic CD8 T cells, neutrophils, and mast cells. While there has been reports of some mast cells expressing CD4, current literature suggests that normal mast cells do not express this particular cell surface marker (58). In addition, the source of IL-17A is most likely not attributed to γδ T cells or ILCs since even though they can express the CD4, these cell types represent a very minor population in the human PBMCs. In the case of γδ T cells, they are a minor population in circulating PBMCs (0.5-5%, compared to 50% of αβ T cells), are more often expressed in epithelial cell-rich compartments like skin, the digestive tract, and reproductive organ mucosa, and mainly exhibit a CD3+CD4-CD8- phenotype (59). ILCs (ILC1, ILC2, and ILC3) do not normally express CD4 either, and even though a subset of CD4+ILC1 cells have been identified, these cells shared more characteristics with Th1 phenotypes in their cytokine production, as opposed to Th17 (60). In addition, like γδ T cells, ILCs are mainly tissue-associated and have low frequency in circulation, such as the PBMC population (61). NK/NKT cells are also lowly expressed in PBMCs compared to T helper cell subsets (62), and in addition, NK cells do not normally express T cell-associated CD3. Collectively, these observations strongly suggest the source of increased IL-17A in PTSD patients is most likely derived from CD4+ Th17 cells, which is supported by a report from Hefele et al. which showed trauma can increase IL-17A specifically in Th17 subsets (63).

Regarding the link between TP53 and miRNA let-7a in our current report, TP53 is known to enhance the maturation of several miRNA already, such as the let-7 family, miR-200c, miR-143, miR-16, miR-145, and miR-21 (64–66). miRNA play a crucial role in the regulation of many biological cellular processes, including CD4+ T cell development, proliferation, differentiation, and function (67). Of particular interest is the fact that let-7a, a member of the let-7 family of miRNAs, was shown to decrease IL-6-dependent Th17 differentiation and production of Th17-specific cytokines in a murine model of Con A-induced hepatitis (68). Let-7 members are known to be direct negative regulators of IL-6 and small guanosine triphosphatases (GTPases) Ras proteins, which play important roles in inducing Th17 cells and IL-17, respectively (69, 70). In cancer cells, TP53 plays an important role in the induction of let-7a (37), and let-7a along with TP53 were shown to regulate Ras in a colorectal cancer cell line (36). This would suggest that let-7a-mediated effects on Th17 function involves a possible indirect relationship between this microRNA and IL-17A, since examination of publicly-available miRNA-target gene prediction websites such as miRdatabase (http://mirdb.org/) and TargetScan (http://www.targetscan.org/) revealed human let-7a did not bind directly to IL-17A transcripts. However, as noted in **Figure 2** of the current report, let-7a can inhibit Ras proteins, which have been implicated in the development of Th17 and production of IL-17 (71). In studies by Zayoud et al., Ras protein inhibitors were found to suppress *in vivo* induction of splenic Th17 cells. In the same report, *in vitro* polarization of Th17 was also perturbed using Ras inhibition. Therefore, it would stand to reason that TP53 downregulation leading to decreased let-7a would prevent directed inhibition of Ras proteins, thereby leading to increased Th17 polarization and IL-17A production in T cells.

Collectively, the current report offers support for the hypothesis that dysregulation of the TP53/let-7a axis could lead to an increased inflammatory Th17 phenotype. This provides evidence in the underreported role of TP53 in PTSD, which is becoming increasingly more prevalent as cases of PTSD are currently on the rise due to the COVID-19 pandemic (72, 73). Despite these exciting findings, the current report does have limitations that must be addressed in future studies. In addition to a relatively overall low sample size, new data generated in the current report contain samples with results more prevalent to both males (sex bias) and African Americans (race/ethnicity bias). Future studies should and must focus now on evaluating these key findings presented in larger and more diverse patient cohorts, with special emphasis on how types of trauma (e.g. combat vs non-combat) and other factors (e.g. sex or race/ethnicity) might affect the TP53/let-7a axis and the subsequent susceptibility to an inflammatory Th17 phenotype in PTSD patients. In addition, and as noted earlier in the discussion, key findings from the current report must be validated specifically in the CD4+ T cell population of patient cohorts as it relates to epigenetic modifications, such as DNA methylation in the TP53 promoter region and let-7a levels. For example, while published reports have shown that TP53 activation can induce let-7a in various human cell lines (74), these findings will need to be further corroborated in human CD4+ T cells by manipulating TP53 (activation and silencing) in this particular cell subset and evaluating how these changes effect let-7a levels. Lastly, based on the more qualitative nature of the MeDIPseq results showing hypermethylation of the TP53 promoter in PTSD patients compared to controls in the current report, ample justification is now provided to examine this observation in a more quantitative assay (e.g. methylation-specific PCR, or MSP) in a larger human subject cohort.

## Data Availability Statement

Publicly available datasets were analyzed in this study. This data can be found here: The datasets generated for this study can be found in the following online repositories: ArrayExpress (E-MTAB-4880) (https://www.ebi.ac.uk/arrayexpress/experiments/E-MTAB-4880/) or Gene Expression Omnibus (GEO; GSE860) (https://www.ncbi.nlm.nih.gov/geo/query/acc.cgi?acc=GSE860).

## Ethics Statement

Procedures to collect human samples presented in this report were approved and reviewed by the Institutional Review Boards (IRBs) from the University of South Carolina, the University of Michigan, and the University of North Carolina-Chapel Hill. The patients/participants provided their written informed consent to participate in this study.

## Author Contributions

The following contributions in the current report acknowledge the roles of the listed authors as follows: PB, MN, and PN contributed to conception and design of the study. PB, MB, XY, JZ, OA, and JG conducted the experiments. JG, MU and AA provided samples and assisted with statistical analysis. PB wrote the first draft of the manuscript. PB, MN, PN, MB, XY, JZ, OA, JG, MU, and AA contributed to manuscript revision and read and approved the submitted version.

## Funding

The work was supported by funding from the National Institutes of Health (NIH) [R01ES019313, R01MH094755, R01AI123947, R01AI129788, P01AT003961, P20GM103641, R01AT006888].

## Conflict of Interest

The authors declare that the research was conducted in the absence of any commercial or financial relationships that could be construed as a potential conflict of interest.

## Publisher’s Note

All claims expressed in this article are solely those of the authors and do not necessarily represent those of their affiliated organizations, or those of the publisher, the editors and the reviewers. Any product that may be evaluated in this article, or claim that may be made by its manufacturer, is not guaranteed or endorsed by the publisher.
